# Cannabis Use in Orthopaedic Surgery: Effects on Fracture Healing, Opioid Requirements, and Clinical Outcomes

**DOI:** 10.7759/cureus.100930

**Published:** 2026-01-06

**Authors:** Hiram E Luigi Martinez, Felix M Rivera Troia, Paola A Babilonia Beltran, Estefanía C Flores Carrasquillo, Rafael Fernandez-Sotero, Rafael Señeriz Ortiz

**Affiliations:** 1 Department of Orthopaedic Surgery, Ponce Health Sciences University, Ponce, PRI

**Keywords:** bone, cannabidiol, cannabinoids, cannabis, cannabis use disorder, complications, fracture healing, opioids, orthopaedic surgery, postoperative pain

## Abstract

Cannabis is commonly used by patients presenting for orthopaedic care, including musculoskeletal pain, sleep disturbances, anxiety, or recreational purposes. Because orthopaedic care depends on predictable bone healing, effective perioperative analgesia, and prevention of postoperative complications, the interaction between cannabis exposure and these domains remains uncertain. This narrative review synthesizes mechanistic, preclinical, and clinical evidence regarding the effects of cannabis and cannabinoid exposure on fracture healing, opioid requirements, and broader clinical outcomes in orthopaedic surgery, and draws on literature gathered via PubMed and Embase supplemented by manual review of references and a structured semantic search using an artificial intelligence assisted platform that screened more than 100 orthopaedic and perioperative studies.

Experimental studies show that the endocannabinoid system influences osteoclast and osteoblast function and that smoked cannabis can impair cancellous bone healing in animal models, while cannabidiol may enhance fracture repair. In addition, observational data in humans associate heavy cannabis use with lower bone mineral density, higher fracture risk, and, in some settings, increased postoperative complications or risks of malunion or nonunion. Across heterogeneous orthopaedic and mixed surgical cohorts, chronic cannabis use is frequently associated with higher postoperative pain scores and greater opioid consumption, while small trials of pharmaceutical cannabinoids demonstrate at most modest opioid-sparing effects that have not translated into clinically important perioperative benefits. Taken together, current evidence does not support routine perioperative cannabis use to reduce opioid requirements and raises concerns regarding bone health and selected postoperative complications, particularly among heavy users, underscoring the need for systematic screening, counseling to avoid inhaled cannabis around fracture fixation or fusion, and reliance on validated multimodal analgesic strategies rather than cannabinoids.

## Introduction and background

Cannabis is among the most widely used psychoactive substances globally, and its legal status has changed rapidly over the past decade. Liberalization of medical and recreational cannabis laws in multiple jurisdictions has increased the prevalence of use among adults, including middle-aged and older individuals who commonly present with degenerative and traumatic musculoskeletal conditions. As a result, orthopaedic surgeons now encounter cannabis users more frequently in elective arthroplasty clinics, sports medicine practices, trauma bays, and perioperative wards with increasing frequency [1-3].

At the same time, orthopaedic care depends on three domains that are potentially influenced by cannabis exposure. First, bone biology and fracture healing determine the success of fixation constructs and whether arthrodesis procedures achieve durable union [4-7]. Second, perioperative pain management and opioid stewardship are central to enhancing recovery pathways and efforts to reduce persistent postsurgical opioid use. Third, postoperative complications, including infection, venous thromboembolism, cardiorespiratory events, and readmission, are key quality metrics and determinants of cost [8-12]. Any intervention that alters these domains has important implications for orthopaedic practice.

At a broad level, cannabinoids exert their effects through the endocannabinoid system, a regulatory network involved in pain modulation, inflammation, and tissue homeostasis. While the molecular mechanisms of this system are complex, its clinical relevance to orthopaedic surgery primarily relates to potential effects on bone metabolism, fracture healing, and perioperative pain. Mechanistically, the endocannabinoid system includes endogenous ligands, classical cannabinoid receptors CB1 and CB2, and nonclassical targets expressed in neural, immune, and skeletal tissues. Through these pathways, exogenous cannabinoids may modulate nociception, inflammation, vascular tone, and osteoblast and osteoclast activity, and other processes relevant to skeletal repair [13].

Against this biologic background, clinicians and patients often ask whether cannabis use is beneficial, neutral, or harmful in the context of fracture healing and orthopaedic surgery. Many patients report subjective pain relief and reduced reliance on prescription opioids when using cannabis products, while others present with cannabis use disorder and polysubstance use associated with complex psychosocial and medical comorbidities. Observational studies have generated seemingly conflicting results, with some large database analyses linking cannabis related diagnostic codes to higher perioperative complication rates and others reporting no increase in risk after controlling for tobacco and other factors [14-17].

The National Academies of Sciences, Engineering, and Medicine concluded that there is substantial evidence that cannabis or cannabinoids can reduce chronic pain in adults, but emphasized that most evidence pertains to neuropathic or mixed chronic pain populations rather than to acute postoperative pain or fracture-related pain [18]. Orthopaedic-specific evidence was limited at the time of that report but has expanded in recent years, particularly within trauma and arthroplasty populations [4,8,11,17,19-21]. Clinicians must now interpret these newer data while navigating divergent patient expectations, regulatory environments, and institutional policies.

This narrative review aims to integrate preclinical and clinical evidence to address three practice-oriented questions. First, how does cannabis exposure influence bone biology and fracture healing, including nonunion and infection rates after fracture fixation or arthrodesis [4,7,8,11,12,17,22-24]. Second, does cannabis use alter postoperative opioid requirements and patient-reported pain after orthopaedic surgery [19,20,25-28]. Third, what are the overall clinical outcomes associated with cannabis use in orthopaedic populations, including medical complications, readmissions, and mortality [8,11,12,14-17,21-23,26,29-31]. By synthesizing available data and highlighting methodological limitations, we seek to provide orthopaedic surgeons with pragmatic guidance for perioperative counseling and decision making while outlining priorities for future research [4,32].

## Review

Methodology

We performed a narrative review rather than a formal systematic review because of the anticipated heterogeneity in study designs, cannabis exposure definitions, and outcome measures, and because the field is evolving rapidly with the frequent addition of new observational datasets and practice guidelines. The review followed the general principles for rigorous narrative synthesis in biomedical research, including transparent description of search strategy, prespecified questions, and explicit consideration of study quality and sources of bias.

Electronic searches of PubMed/MEDLINE were conducted from database inception through October 2025 using combinations of keywords and medical subject headings (MeSH) related to cannabis, marijuana, cannabinoids, cannabidiol, tetrahydrocannabinol, orthopaedic surgery, fracture, bone healing, arthroplasty, trauma, postoperative pain, and opioid use. Reference lists of relevant narrative reviews, systematic reviews, and primary orthopaedic studies were manually screened to identify additional articles.

To complement traditional database searches and reduce the likelihood of missing studies using nonstandard terminology, we additionally employed artificial intelligence-assisted semantic searches using Semantic Scholar (Allen Institute for AI, Seattle, WA, USA) and OpenAlex (OurResearch, Sanford, NC, USA). These searches yielded several hundred candidate publications related to cannabis use and orthopaedic surgery.

Eligible studies included adult human studies involving surgically managed fractures or orthopaedic procedures that documented cannabis use or cannabinoid exposure and reported at least one relevant outcome related to bone healing, opioid consumption, pain, functional recovery, or postoperative complications. Case reports, case series with fewer than 10 patients, non-orthopaedic procedures, and studies lacking clear definitions of cannabis exposure were excluded. Preclinical and translational studies examining cannabinoid signalling in bone biology or fracture repair were identified through targeted searches and citation tracking.

For each included clinical study, we extracted key methodological features, including study design, population characteristics, definition and assessment of cannabis exposure, type of orthopaedic procedure, reported outcomes, and analytic approach. Particular attention was paid to whether studies distinguished cannabis-only use from combined cannabis and tobacco use and whether analyses accounted for relevant confounders such as age, comorbidities, injury severity, and other substance use.

Given the predominance of retrospective observational designs, we did not perform formal quantitative pooling or risk-of-bias scoring for individual studies. Instead, we qualitatively appraised potential sources of bias, including exposure misclassification, outcome misclassification, unmeasured confounding, selection bias, and missing data. Evidence was synthesised narratively rather than quantitatively, as quantitative synthesis, including meta-analysis or meta-regression, was not feasible due to substantial heterogeneity in study designs, exposure definitions, outcome measures, and reporting methods, and was organised into three domains: (1) bone biology and fracture healing, (2) postoperative pain and opioid requirements, and (3) perioperative and clinical outcomes [33]. No proprietary scales, scoring systems, or questionnaires were applied in this narrative review, and no permissions or licences were required.

Endocannabinoid signaling and bone biology

The endocannabinoid system comprises endogenous ligands, receptors, and enzymes that collectively regulate synaptic transmission, neuromodulation, metabolic homeostasis, and immune function. The cannabinoid type 1 (CB1) receptors are expressed primarily in the central and peripheral nervous systems but are also present in sympathetic nerve terminals within bone, whereas cannabinoid type 2 (CB2) receptors are expressed predominantly on immune cells and in skeletal tissues. Endocannabinoids such as anandamide and 2-arachidonoylglycerol are synthesised on demand from membrane lipid precursors and degraded by fatty acid amide hydrolase and monoacylglycerol lipase, respectively. Exogenous phytocannabinoids, including delta-9-tetrahydrocannabinol and cannabidiol, as well as synthetic cannabinoids, interact with these receptors and with nonclassical targets such as GPR55 and transient receptor potential channels [13].

Seminal murine studies have demonstrated that CB1 and CB2 signalling exert distinct and sometimes opposing effects on bone mass and architecture. The CB2-deficient mice develop an age-related osteoporosis phenotype characterised by reduced trabecular bone volume and increased bone turnover, and CB2-selective agonists stimulate osteoblast differentiation and attenuate ovariectomy-induced bone loss in rodent models. In contrast, global CB1 knockout in young mice leads to increased peak trabecular bone mass due to reduced osteoclast recruitment and bone resorption, yet CB1 signalling appears to protect against age-related bone loss by regulating the lineage commitment of bone marrow stromal cells toward osteoblast rather than adipocyte differentiation [33-35].

Additional work has highlighted interactions between cannabinoid signalling and the sympathetic nervous system. The CB1 receptors in sympathetic terminals modulate norepinephrine release and thereby influence osteoblast activity and bone formation. Loss of CB1 signalling in ageing mice has been associated with accelerated bone loss, increased marrow adiposity, and impaired bone formation, reinforcing the concept that the timing and cellular context of cannabinoid receptor modulation are critical determinants of skeletal effects [36-38].

Translational studies extend these mechanistic insights to fracture repair. In a rat femoral fracture model, cannabidiol improved the biomechanical properties of the healing callus and enhanced collagen crosslinking through upregulation of lysyl hydroxylase activity, whereas delta-9-tetrahydrocannabinol alone did not confer these benefits and may have attenuated them. Other preclinical work indicates that high-dose inhaled cannabis smoke impairs bone formation around titanium implants, with reduced trabecular bone fill and bone-to-implant contact in rat tibial metaphyseal models. Taken together, these data suggest that cannabinoid signalling is intimately involved in skeletal homeostasis and that specific ligands and routes of exposure may have divergent effects on bone remodelling and fracture repair [39,40].

Importantly, these preclinical observations cannot be extrapolated directly to human cannabis use patterns. Rodent models typically involve controlled dosing of isolated cannabinoids or standardised smoke exposures, whereas human users consume heterogeneous products that vary widely in tetrahydrocannabinol concentration, cannabidiol content, route of administration, and frequency of use [39,40]. Nevertheless, the biologic plausibility established by mechanistic and animal studies provides a foundation for interpreting clinical observations regarding bone mineral density, fracture risk, and union outcomes in cannabis-exposed patients [35,41-43]. Selective preclinical and translational studies examining cannabinoid signalling in bone biology are summarised in Table 1.

**Table 1:** Selected preclinical and translational studies of cannabinoid signaling and bone biology

Study/model	Exposure or intervention	Key skeletal outcome	Direction of effect
Ofek et al. [33] CB2 knockout mice	Genetic deletion of the CB2 receptor	Age-related trabecular bone loss and high turnover osteoporosis phenotype	Harm (loss of osteoprotective CB2 signaling)
Idris et al. [35] CB1 knockout mice	Genetic deletion of the CB1 receptor	Higher peak trabecular bone mass in young mice, but accelerated age-related bone loss	Mixed (context-dependent effects on bone)
Kogan et al. [39] rat femoral fracture model	Cannabidiol versus delta 9 tetrahydrocannabinol versus vehicle	Improved callus strength and collagen crosslinking with cannabidiol; no benefit with tetrahydrocannabinol alone	Benefit from cannabidiol
Filho et al. [40] rat tibial implant model	Chronic inhalation of cannabis smoke	Reduced trabecular bone fill and bone-to-implant contact around titanium implants	Harm (impaired cancellous healing)

Patterns of cannabis use in orthopaedic populations

Descriptions of cannabis exposure in orthopaedic studies vary considerably, which complicates the synthesis and translation of findings. Most large database analyses identify cannabis users through International Classification of Diseases diagnostic codes that capture cannabis use, abuse, or dependence and that may preferentially identify individuals with cannabis use disorder rather than casual or intermittent users. Other studies rely on self-reported current use, structured questionnaires, or urine toxicology screening at hospital admission. A smaller subset distinguishes medical cannabis authorisation from recreational use or documents exposure to prescribed cannabinoid medications such as dronabinol or nabiximols [14,31,42-46].

Across orthopaedic cohorts, cannabis users tend to be younger and more often male compared with nonusers and exhibit higher rates of concomitant tobacco use, alcohol misuse, and other substance use. Surveys of orthopaedic trauma and hand surgery patients in North America report lifetime cannabis use in more than half of respondents and current use in approximately one quarter, with most current users consuming smoked or vaporised products several times per week or daily. Pain relief, improved sleep, and anxiety reduction are among the most frequently cited reasons for use. In many datasets, dual use of cannabis and tobacco is common, and polysubstance exposure is prevalent in trauma populations, in which urine toxicology screens frequently detect amphetamines, benzodiazepines, opioids, or cocaine in addition to tetrahydrocannabinol [1-3,44,47]. 

These patterns are clinically important because tobacco use and polysubstance exposure are independently associated with impaired wound healing, higher infection rates, and worse functional outcomes after orthopaedic surgery. Failure to differentiate cannabis-only users from individuals who co-use tobacco or other substances can therefore lead to overestimation of cannabis-specific risk. Several orthopaedic studies that have specifically separated these groups report that cannabis-only use is not associated with increased risk of major complications, whereas combined cannabis and tobacco use confers the highest risk of adverse outcomes [8,11,12,17,22-24,48].

Another limitation of current literature is the lack of granular information on dose, potency, and route of administration. Very few clinical studies report estimated tetrahydrocannabinol or cannabidiol content, quantify daily or weekly use in standardised units, or differentiate smoked and vaporised products from oral, sublingual, or topical formulations. These details are critical because inhaled combustion products may impair vascular and immune responses in ways that oral cannabinoids do not, and because cannabidiol-dominant preparations may have different skeletal and analgesic effects compared with tetrahydrocannabinol-dominant products [4,7,32,39,40,49,50].

Finally, orthopaedic literature has only recently begun distinguishing cannabis use disorder from non-disordered use. Population-based analyses in general surgical cohorts indicate that cannabis use disorder is associated with modestly increased composite perioperative morbidity and mortality, although these associations are attenuated when adjusted for comorbid psychiatric illness and polysubstance use [30,31]. Whether similar patterns hold in orthopaedic-specific populations remains an active area of investigation [4,11,22,51]. These mechanistic observations provide a framework for interpreting clinical studies evaluating fracture healing and union outcomes in cannabis-exposed patients.

Cannabis and fracture healing

Fracture healing is a complex process requiring coordinated inflammatory, reparative, and remodelling phases and is influenced by systemic factors such as age, comorbidities, smoking status, and nutritional state, as well as local mechanical and biologic factors. Understanding whether cannabis exposure alters this process is a priority for orthopaedic surgeons who are responsible for fracture fixation and arthrodesis procedures [4,7].

Human data regarding cannabis and bone health can be considered in three tiers: (1) bone mineral density and fracture risk, (2) healing outcomes after upper extremity fractures, and (3) healing outcomes after lower extremity and foot and ankle fractures. Additional insights arise from maxillofacial and spine cohorts [4,8,11,12,17,21-24,41,42,51].

Cross-sectional observational studies have associated heavy cannabis use with lower bone mineral density and higher fracture risk [41,42]. In a population-based cohort study, Sophocleous et al. (2017) reported that heavy cannabis use was associated with reduced hip and spine bone mineral density and increased fracture prevalence compared with matched controls, even after adjusting for body mass index and alcohol use [41]. A broader rheumatology-oriented review concluded that chronic, high-dose cannabis exposure appears to be a risk factor for reduced bone mineral density and osteoporotic fractures, although the underlying mechanisms remain incompletely understood [42]. These findings align with preclinical evidence that disruption of CB2 signalling accelerates age-related bone loss but do not directly address fracture healing after orthopaedic procedures [35,36,52].

Observed differences across anatomic regions likely reflect heterogeneity in study populations, exposure definitions, and study methodologies rather than established biologic differences in fracture healing response. Upper extremity fracture cohorts provide more direct insights into union and malunion. In large database studies of distal radius open reduction and internal fixation, Lyvesey et al. (2023) reported higher rates of postoperative infection and malunion among cannabis users, with the greatest risk observed in patients with combined cannabis and tobacco use [8]. A national cohort study by Heath et al. (2022) of patients undergoing wrist fracture fixation, including distal radius and carpal fractures, found that cannabis users experienced higher ninety-day medical complications and higher rates of surgical site infection and nonunion in the subset with scaphoid fractures. The series reported that patients with distal radius fractures who used both cannabis and tobacco had the greatest risk of nonunion, whereas cannabis-only users had a more modest increase in malunion risk [4]. Evidence for metacarpal fractures and other hand injuries is emerging. A recent database study of metacarpal fracture fixation demonstrated that cannabis and tobacco use were each associated with increased surgical complications, with the highest risk observed in dual users [5]. However, the absolute rates of nonunion were low, and radiographic criteria for union were not standardised across institutions, limiting definitive conclusions regarding cannabis-specific effects on upper extremity bone healing [4].

Lower extremity and long bone data are more heterogeneous. In a multicentre cohort of tibial shaft fractures treated with intramedullary nailing, marijuana users exhibited higher rates of 90-day surgical complications and deep infection in unadjusted analyses, yet marijuana use was not an independent predictor of surgical complications or deep infection after adjustment for tobacco use, open fracture status, and comorbidities [11]. Similarly, some database studies of ankle fracture fixation have suggested increased infection and readmission rates among patients with cannabis-related diagnostic codes, while others that distinguish cannabis-only from tobacco users have found no significant differences in union or major complications between cannabis-only users and nonusers [17,22,23].

Foot and ankle arthrodesis cohorts provide additional nuance. In a large database study, cannabis use was associated with higher 90-day medical complications and resource utilization [23]. In contrast, a more recent analysis specifically examined cannabis-only users undergoing ankle and hindfoot arthrodesis and reported no increased risk of 90-day or two-year complications compared with nonusers, whereas tobacco use was associated with higher nonunion and infection rates [22]. These findings underscore the importance of disentangling cannabis-specific effects from tobacco-related and comorbidity-related risks [12,14].

Beyond the appendicular skeleton, facial fracture series highlight the interplay between cannabis and tobacco. In a cohort of patients undergoing mandible and midface fracture surgery, patterns of postoperative complications differed by substance use profile. Tobacco and combined cannabis-tobacco users experienced higher rates of surgical site infections, abscess formation, and reoperation, whereas cannabis-only users had complication rates closer to those of nonusers [24]. Once again, confounding by tobacco appears to be a dominant driver of risk in many settings [12,24].

Taken together, contemporary evidence suggests that heavy cannabis exposure is associated with lower bone mineral density and that certain fracture cohorts, particularly distal radius, wrist, and scaphoid fractures, show higher rates of infection, malunion, or nonunion among cannabis users. However, when rigorous adjustments for tobacco and comorbidities are applied, cannabis-only use often shows neutral or modest associations with union outcomes, and long bone studies do not consistently demonstrate an independent effect on nonunion [8,11,17,22,23,41,42,48]. These mixed findings must be interpreted in the context of significant methodological limitations, including retrospective designs, reliance on diagnostic codes rather than detailed exposure assessment, and heterogeneity in radiographic and clinical definitions of union [6,8,11,14,17,22,23,46,48,53,54].

From a practical standpoint, orthopaedic surgeons should recognise that cannabis use may cluster with other risk factors for impaired fracture healing, particularly tobacco use, poor nutrition, and psychosocial stressors. Careful assessment of global risk, counselling on cessation of inhaled products, optimisation of modifiable factors, and close radiographic follow-up are prudent for cannabis-using patients undergoing fracture fixation or arthrodesis, especially in anatomic sites where even small decrements in union probability have important functional consequences [14,16,19,23,32,54]. Key clinical studies evaluating cannabis exposure and fracture or fusion outcomes across orthopaedic populations are presented in Table 2. 

**Table 2:** Representative clinical studies of cannabis exposure and fracture or fusion outcomes Note: Where abstracts did not provide event percentages, the table reports the statistical significance/effect estimates that were provided. ORIF: Open reduction internal fixation; OR: Odds ratio; CCI: Charlson comorbidity index; AKI: Acute kidney injury; DVT: Deep venous thrombosis; MI: Myocardial infarction; UTI: Urinary tract infection

Study and population	Cannabis exposure definition	Sample size (exposed vs comparator; total)	Primary outcome(s)	Key quantitative results (selected)
Livesey et al. [8] (national claims database): distal radius fracture open reduction internal fixation (ORIF) (2015-2020)	Tobacco-only vs cannabis-only vs tobacco+cannabis vs neither (“control”); smoking defined ≥1 year pre- and post-op	Total N=970,747. Tobacco: 86,941; cannabis: 898; tobacco+cannabis: 9,842; control: 747,892 (study also reports sampling 20,000 from tobacco and control for regression model)	One-year infection, nonunion, malunion	Nonunion: tobacco+cannabis 5.0% (higher vs tobacco-only or cannabis-only; p<0.001 infection cannabis-only tobacco-only tobacco malunion>
Kishan et al. [5] (insurance claims): metacarpal fracture fixation (2010-2022)	Diagnosed cannabis use disorder/dependence/addiction vs tobacco history vs neither substance (“control”); propensity matched for age/sex/Charlson comorbidity index (CCI)	Total N=80,787; cannabis users: 5,043 (6.7%); comparators = tobacco users + control group (numbers vary by matching set)	90-day medical complications; six‑month surgical complications	90 days (vs control): higher incidence of acute kidney injury (AKI), cardiac arrest, deep vein thrombosis (DVT), hypoglycemia, myocardial infarction (MI), pneumonia, sepsis, stroke, and urinary tract infection (UTI) (all p<0.01) within 90 days after surgery. After matching (cannabis vs control): higher incidence of nerve injury (p<0.01), fracture nonunion (p=0.04), and fracture malunion (p=0.002). Compared with tobacco users: lower incidence of pneumonia (p=0.002), UTI (p<0.01), and hypoglycemia (p=0.03) within 90 days
Maxson et al. [1] (two level-I trauma centers): tibia shaft fracture fixation (2014-2022)	Current marijuana use by self-report or urine toxicology + at presentation	Total N=388; marijuana users 96 (25%) vs nonusers 292	90-day thromboembolic & surgical complications; deep infection; union outcomes (≥6 months follow-up)	90-day surgical complications: 11.5% vs 4.8% (p=0.030). Deep infection: 8.3% vs 2.1% (p=0.008). Multivariable: marijuana use was not independently associated with any 90‑day surgical complication (odds ratio (OR) 2.01; 95% CI 0.83–4.84) or deep infection (OR 2.97; 95% CI 0.95–9.25).
Holle et al. [2] (claims database): ankle/hindfoot arthrodesis	Separate matched analyses: cannabis-only vs control; tobacco-only vs control; tobacco+cannabis vs control; tobacco+cannabis vs tobacco	Total N=61,705. Matched cohorts: cannabis-only 380 vs 1,520 control; tobacco-only 25,114 vs 33,250 control; tobacco+cannabis 2,294 vs 8,776 control; tobacco+cannabis 2,291 vs tobacco 8,320	90-day medical outcomes; two-year surgical outcomes (including infection/nonunion)	Cannabis-only: no significant increase in postoperative complications vs control. Tobacco-only vs control: nonunion 15.5% vs 11.1% (OR 1.33); infection 16.4% vs 11.8% (OR 1.24). Tobacco+cannabis vs control: nonunion 19.6% vs 10.8% (OR 1.90). Tobacco+cannabis vs tobacco: ED visits 29.7% vs 20.9% (OR 1.45); nonunion 19.6% vs 16.4% (OR 1.19).

Cannabis, postoperative pain, and opioid requirements

Beyond skeletal biology, cannabinoid signalling may influence postoperative pain perception and analgesic requirements, prompting investigation in orthopaedic surgical populations. The relationship between cannabis use and postoperative pain is of particular interest to orthopaedic surgeons who seek to provide effective analgesia while minimising opioid exposure. Patients frequently report using cannabis products with the expectation that they will decrease opioid requirements, and some clinicians question whether cannabis should be incorporated into multimodal analgesia regimens [2,3,27,28,47].

Current evidence distinguishes between chronic preoperative cannabis use and short-term therapeutic administration of standardised cannabinoid preparations in the perioperative setting. In orthopaedic and broader surgical cohorts, chronic cannabis use is generally associated with higher, rather than lower, postoperative pain scores and opioid consumption. In a propensity-matched study of major orthopaedic procedures, preoperative cannabinoid users reported significantly higher pain scores at rest and with movement during the first 36 hours after surgery, with nearly twice the odds of experiencing moderate to severe pain compared with nonusers, despite receiving similar or greater amounts of opioid analgesia. Sleep disruption and dissatisfaction with pain control were also more common among cannabis users in this cohort [28].

Similar findings have been observed in mixed-age surgical populations. In a large perioperative cohort, Sajdeya et al. (2024), who used natural language processing and structured data to identify perioperative cannabis users among older adults, found that users had higher dynamic pain scores and consumed more oral morphine equivalents in the first 24 hours after surgery than matched nonusers [25]. In another multicentre cohort including orthopaedic and general surgical procedures, self-reported cannabis use after discharge was associated with higher cumulative opioid pill counts, greater likelihood of reporting moderate to severe surgical site pain at one week and one month, lower global satisfaction with surgery, and worse patient-reported quality of life [27].

Trauma cohorts further support this hyperalgesic pattern. Among patients with musculoskeletal injuries treated at level I trauma centres, self-reported marijuana use during recovery was associated with higher total prescribed opioid morphine milligram equivalents and longer duration of opioid use compared with patients who never used marijuana, even after adjustment for injury severity and psychosocial factors [19]. Similarly, in a trauma population where urine toxicology was used to identify tetrahydrocannabinol-positive patients, cannabis exposure was associated with substantially higher inpatient opioid utilisation relative to tetrahydrocannabinol-negative controls [26]. These findings align with preclinical evidence that chronic cannabinoid exposure can induce tolerance and hyperalgesia through downregulation of CB1 receptors and crosstalk with endogenous opioid systems [13,55].

In contrast, evidence for an opioid-sparing effect of therapeutically administered cannabinoids comes primarily from small trials in non-orthopaedic settings and is mixed. A matched pilot study of dronabinol for acute pain after traumatic injury suggested that adjunctive dronabinol might reduce inpatient opioid requirements compared with standard care, particularly among patients with prior marijuana use; however, the sample size was limited, and findings have not yet been replicated in orthopaedic-specific trials [56]. Systematic reviews of randomised trials involving tetrahydrocannabinol, cannabidiol, and combination products for acute postoperative pain across surgical disciplines have concluded that cannabinoids provide, at best, modest incremental analgesia and do not consistently reduce opioid consumption in clinically meaningful ways [57-59]. 

Scoping reviews restricted to orthopaedic surgery underscore the paucity of high-quality experimental evidence. In a recent review of experimental studies examining cannabis and cannabidiol for postoperative orthopaedic pain, only a small number of randomised or quasi-experimental trials were identified, with heterogeneity in formulations, dosing regimens, surgical procedures, and endpoints [50,56]. Some trials of balanced tetrahydrocannabinol-cannabidiol formulations reported small reductions in pain scores or opioid use, whereas others did not demonstrate significant benefits [50,56]. Adverse effects, including dizziness, dysphoria, and cognitive impairment, were more common with tetrahydrocannabinol-dominant preparations [46,59].

An additional complexity is the discrepancy between patient perceptions and objective outcomes. Surveys of orthopaedic patients indicate that a majority believe cannabis is effective for pain control and perceive that cannabis use during recovery reduces their opioid intake [2,3,47]. However, when opioid prescriptions and consumption are measured quantitatively, cannabis users frequently exhibit equal or greater opioid exposure compared with nonusers [19,20,25-28]. This mismatch suggests that subjective impressions of benefit may be influenced by mood-altering or anxiolytic effects rather than true analgesia and reinforces the need for cautious interpretation of patient self-report when counselling about cannabis as an analgesic adjunct [46,57,59].

On balance, contemporary evidence does not support routine recommendation of cannabis or over-the-counter cannabinoid products as part of standard multimodal analgesia pathways for orthopaedic surgery. Clinicians should instead prioritise proven strategies such as regional anaesthesia, acetaminophen, nonsteroidal anti-inflammatory drugs when appropriate, gabapentinoids in selected patients, and non-pharmacologic modalities. For patients who choose to continue cannabis use around the time of surgery, it is reasonable to counsel that available data do not demonstrate meaningful reductions in opioid requirements and that heavier use may actually be associated with worse pain and higher opioid consumption in the acute postoperative period [19,20,25-28,46,50,56-59]. Selected studies assessing the relationship between cannabis use, postoperative pain, and opioid requirements are outlined in Table 3.

**Table 3:** Selected studies of cannabis exposure, postoperative pain, and opioid requirements DVPRS: Defense and Veterans Pain Rating Scale; OME: Oral morphine equivalents; MME: Morphine milligram equivalents; OR: Odds ratio; aOR: Adjusted odds ratio;  QoL: Quality of life; MSQC: Michigan Surgical Quality Collaborative; NLP: Natural language processing

Study and population	Cannabis exposure definition	Sample size (exposed vs comparator; total)	Primary outcome(s)	Key quantitative results (selected)
Liu et al. [28] (major orthopaedic surgery; retrospective cohort with propensity matching)	Preoperative cannabinoid use (recreational or medical) compared to nonuse	Total N=3,793; cannabinoid users 155. After matching: 155 users vs 155 nonusers	Early postoperative pain (≤36h): pain at rest and movement; incidence of moderate-to-severe pain; sleep interruption	Pain at rest (median (IQR)): 5.0 (3.0–6.1) vs 3.0 (2.0–5.5) (p=0.010). Pain with movement: 8.0 (6.0–9.0) vs 7.0 (3.5–8.5) (p=0.003). Moderate to severe pain at rest: 62.3% vs 45.5% (odds ratio (OR) 1.98; 95% CI 1.25–3.14; p=0.004). Moderate–severe pain with movement: 85.7% vs 75.2% (OR 1.98; 95% CI 1.10–3.57; p=0.021).
Sajdeya et al. [25] (older adults ≥65; propensity matched cohort; ≥24h stay)	Cannabis use within 60 days pre-op (natural language processing (NLP) + structured EHR), compared to nonuse	Matched cohort N=504: cannabis 126 vs nonusers 378	Average pain (DVPRS) and total opioid dose (oral morphine equivalents (OME)) within 24 hours postop	Defense and Veterans Pain Rating Scale (DVPRS) (median (IQR)): 4.68 (2.71–5.96) vs 3.88 (2.33–5.17), difference 0.80 (95% CL 0.19–1.36; p=0.01). Total OME: 42.50 mg (15–60) vs 30.00 mg (7.5–60), difference 12.5 mg (95% CL 3.8–21.2; p=0.02).
Bicket et al. [27] (Michigan Surgical Quality Collaborative (MSQC) registry; 16 procedures; 69 hospitals; postop surveys)	Patient-reported cannabis (CBD/marijuana) use for pain after discharge, compared to nonuse	Total N=11,314; cannabis users 581 (5.1%) vs nonusers 10,733	Postdischarge opioid consumption; patient-reported pain and satisfaction outcomes	Adjusted models: +1.0 opioid pills (95% CI 0.4–1.5) in cannabis users. Higher odds of moderate-to-severe surgical site pain at one week (adjusted odds ratio (aOR) 1.7; 95% CI 1.4–2.1) and one month (aOR 2.1; 95% CI 1.7–2.7). Lower high satisfaction (72.1% vs 82.6%), best quality of life (QoL) (46.7% vs 63.0%), and no regret (87.6% vs 92.7%) (all p<0.01)
Bhashyam et al. [19] (traumatic MSK injury; self-reported marijuana use)	Self-reported marijuana use status (never/prior only/during recovery)	Total N=500; never 39.8%, prior-only 46.4%, during recovery 13.8% (~69 pts)	Persistent opioid use; total prescribed opioids (morphine milligram equivalents (MME)); duration of opioid use	Persistent opioid use rate 17.6% to 25.9%, and not associated with marijuana use during recovery. Marijuana use during recovery was associated with +343 MME total prescribed opioids (95% CI 87-600; p=0.029) and +12.5 days duration (95% CI 3.4–21.5; p=0.027) vs never-users.

Cannabis use, perioperative complications, and clinical outcomes 

Beyond pain and fracture healing, orthopaedic studies have examined associations between cannabis exposure and a spectrum of perioperative medical and surgical complications. These include pulmonary and cardiovascular events, venous thromboembolism, infections, wound complications, reoperations, readmissions, and mortality [8,11,12,14-17,21-23,26,29-31].

Several large administrative database analyses in orthopaedic populations have reported higher rates of postoperative complications among patients with documented cannabis use or cannabis use disorder. In national datasets of patients undergoing ankle fracture fixation, distal radius fixation, and foot and ankle procedures, cannabis-related diagnostic codes were associated with increased 90-day medical complications such as pneumonia, deep vein thrombosis, pulmonary embolism, myocardial infarction, stroke, acute kidney injury, urinary tract infection, sepsis, and hypoglycemia. The magnitude of risk was often substantial, with two- to three-fold increases in pneumonia and urinary tract infection rates compared with nonusers [8,14,17,21-23,31].

However, not all orthopaedic studies have replicated these findings, and those incorporating more rigorous adjustment for tobacco use and other confounders frequently report attenuated or null associations. In a study of ankle fracture open reduction and internal fixation, cannabis-only users did not exhibit higher odds of 90-day adverse events compared with nonusers, whereas tobacco use and combined cannabis-tobacco use were associated with increased complications. Similarly, a retrospective cohort evaluating marijuana smoking and surgical site infection after orthopaedic procedures found no significant increase in infection risk among marijuana smokers after adjusting for confounders. These observations support the notion that at least some of the complication signals attributed to cannabis in large databases may actually reflect concomitant tobacco use and polysubstance exposure rather than isolated pharmacologic effects of cannabinoids [12,17,22-23].

Spine and arthroplasty cohorts offer additional insights. In lumbar fusion patients, preoperative marijuana use has been associated with higher reoperation rates and longer hospital length of stay, and cannabis use disorder has been linked to increased perioperative morbidity and mortality after major elective inpatient surgery in general surgical cohorts [30,51]. A meta-analysis of hip and knee arthroplasty populations with cannabis use disorder demonstrated higher odds of postoperative infection and implant-related complications, longer length of stay, and higher costs compared with nonusers, although residual confounding is likely. In contrast, a multicenter spine surgery series reported that cannabis use was not independently associated with higher perioperative complication rates after adjustment for comorbidities, underscoring heterogeneity in populations and analytic approaches [14-26,21,31].

Mortality outcomes have garnered particular attention. An analysis of more than nine million orthopaedic procedures, including total hip and knee arthroplasty, shoulder arthroplasty, spine fusion, and femur fixation, found that patients with documented marijuana use had lower in-hospital mortality than matched nonusers, despite similar or [29]. The authors hypothesized that this paradox might reflect a 'healthy user' effect, in which cannabis users were younger and had fewer severe comorbidities, or that tetrahydrocannabinol-mediated cardiometabolic effects might confer some protection in selected contexts. Given the observational nature of the data, causal inference is not possible, and these findings should not be interpreted as evidence that cannabis is protective in orthopaedic surgery [14,31].

Anesthesia-related effects of cannabis exposure are also relevant for orthopaedic care. In a retrospective cohort of adult patients undergoing urgent orthopaedic trauma surgery, tetrahydrocannabinol-positive patients on urine toxicology testing were less likely to require intraoperative vasopressor support and had higher mean arterial pressures under general anesthesia than tetrahydrocannabinol-negative patients [44]. The authors proposed that chronic cannabinoid exposure might lead to downregulation of CB1 receptors and altered autonomic tone that attenuate the hypotensive effects of anesthetic agents [13]. While intriguing, these findings should be interpreted cautiously and do not support deliberate perioperative cannabis use, particularly given concerns about airway reactivity, tachycardia, and hemodynamic liability with acute intoxication [13,44-45].

Overall, the literature on perioperative complications by Kwaczala et al. (2025) suggests that cannabis use disorder and heavy cannabis exposure, especially when combined with tobacco and other substances, mark a higher-risk phenotype in orthopaedic populations [14,17,22-23,31,51,53]. Some of this risk likely reflects social determinants of health, comorbid psychiatric illness, and polysubstance use rather than direct pharmacologic effects [14,31]. Nevertheless, awareness of these associations should prompt careful preoperative assessment, optimization of comorbidities, and multidisciplinary perioperative planning for patients with cannabis use disorder who are undergoing major orthopaedic procedures [14,31,53].

Special populations and contextual factors

The impact of cannabis exposure on orthopaedic outcomes is likely to vary across patient subgroups and clinical contexts. Age, pattern of use, indication for use, and the presence of cannabis use disorder all influence risk profiles and must be considered when interpreting the literature and counseling individual patients [14,32,45].

Older adults represent an increasingly important population as cannabis use rises among individuals in their 60s and 70s. In a propensity-matched analysis of adults aged 65 years and older undergoing a variety of surgical procedures, perioperative cannabis users had higher postoperative pain scores and greater opioid consumption in the first 24 hours after surgery compared with matched nonusers [25]. Age-related changes in pharmacokinetics and pharmacodynamics, as well as the higher prevalence of cardiovascular and cerebrovascular disease, may heighten susceptibility to adverse hemodynamic and cognitive effects of cannabinoids in this group [13,44,45]. Clinicians should be particularly vigilant when older cannabis users present for orthopaedic surgery, emphasizing preoperative counseling, cautious titration of sedatives and opioids, and close postoperative monitoring [44,45].

Trauma versus elective surgery contexts also matter. Trauma patients may be more likely to have polysubstance exposure, unstable social situations, and limited health-care engagement, factors that can independently worsen outcomes after musculoskeletal injury [14,31,45]. In some trauma cohorts, tetrahydrocannabinol positivity on admission toxicology has correlated with higher inpatient opioid requirements and longer hospital stays, whereas in elective hand or foot and ankle surgery populations, cannabis use has not always been independently associated with increased complications when confounders are addressed [19,22,26,48]. These differences highlight the importance of considering the broader clinical scenario rather than focusing solely on cannabis exposure [14,31].

Distinguishing cannabis use disorder from intermittent or medical use is another crucial consideration. Cannabis use disorder encompasses a pattern of use associated with impaired control, social and occupational problems, risky use, and physiologic adaptation, and it frequently coexists with mood disorders, anxiety, and other substance use disorders [14,45]. Large perioperative cohorts have demonstrated that patients with cannabis use disorder experience higher composite morbidity and mortality after major elective inpatient surgery, even after extensive adjustment for comorbidities [14-16,31]. In orthopaedic settings, patients with cannabis use disorder may be less adherent to postoperative weight-bearing restrictions, more likely to miss follow-up visits, and more prone to poor nutritional and self-care behaviors, all of which can influence fracture healing and rehabilitation irrespective of direct pharmacologic effects of cannabinoids [14,31,45].

Finally, emerging data suggest that cannabidiol-dominant or CB2-selective interventions might have different risk-benefit profiles than tetrahydrocannabinol-dominant products. Preclinical studies support a potential osteoanabolic and pro-repair role for cannabidiol, and pilot clinical trials are underway to evaluate cannabidiol as an adjunct for fracture-related pain and inflammation [38,39,42,60]. Until these trials are completed, it remains premature to recommend isolated cannabidiol for fracture healing outside of a research setting [4,39,60]. Clinicians should caution patients that over-the-counter cannabidiol products are highly variable in purity and dosage and that their safety and efficacy in orthopaedic populations are not well established [45-46].

Practical perioperative guidance for orthopaedic surgeons 

Given the limitations and heterogeneity of existing evidence, perioperative management of orthopaedic patients who use cannabis should focus on careful assessment, risk stratification, and incorporation of current best practices in multimodal analgesia and complication prevention rather than blanket prohibitions or endorsements of cannabis use [4,14,17,22,28,31,45,46,51]. First, clinicians should implement routine, nonjudgmental screening for cannabis use as part of the preoperative evaluation. Key elements include timing of last use, frequency and duration of use, typical dose or amount, route of administration, reasons for use, and a history of cannabis use disorder or cannabis-related problems [14,45]. Screening tools for substance use disorders can be adapted for this purpose, and specific inquiry about concomitant tobacco, alcohol, and other drug use is essential [14,31,45]. Documenting cannabis exposure in the medical record facilitates multidisciplinary communication among surgeons, anesthesiologists, internists, and nursing staff [14,45].

Second, elective surgery should be deferred in patients who present with signs of acute cannabis intoxication, such as altered mental status, tachycardia, or hemodynamic instability, because of concerns about impaired consent capacity, aspiration risk, and unpredictable anesthetic responses [44,45]. For patients who use smoked or vaporized cannabis regularly, it is reasonable to counsel abstinence for at least several days before surgery to reduce airway reactivity and carbon-monoxide-related effects and to encourage continued abstinence during the early postoperative period of fracture healing or arthrodesis [40,45,53]. The optimal duration of abstinence is unknown, but recommendations analogous to those for tobacco cessation, if feasible, are biologically plausible and unlikely to cause harm [53].

Third, perioperative analgesia should emphasize evidence-based multimodal regimens rather than rely on cannabis or cannabinoids as primary analgesics. For most orthopaedic procedures, a combination of regional anesthesia, acetaminophen, nonsteroidal anti-inflammatory drugs, or cyclooxygenase (COX)-2-selective inhibitors when not contraindicated, and judicious short-course opioids provides effective pain control and minimizes opioid-related adverse events. Gabapentinoids, ketamine, and nonpharmacologic modalities such as cryotherapy, early mobilization, and cognitive-behavioral strategies can be considered in selected patients. If patients choose to continue cannabis use, clinicians should monitor for additive sedative effects when combined with opioids or benzodiazepines and should avoid dose escalation of opioids solely based on patient expectation that cannabis will allow higher opioid doses without additional risk [28,44,45].

Fourth, surgeons should consider cannabis use in the context of other risk factors when planning postoperative follow-up and rehabilitation. Patients with heavy cannabis use or cannabis use disorder, particularly those who also smoke tobacco, may benefit from closer radiographic surveillance for delayed union or nonunion, more frequent wound checks, and proactive coordination with primary care, mental health, or addiction specialists. Early identification of infection or hardware failure is especially important in fractures of the wrist, scaphoid, tibia, and foot and ankle, where revision surgery carries substantial morbidity [8,11,17,22-24,53].

Finally, orthopaedic teams should engage in shared decision-making with patients who use cannabis, providing balanced information about known risks and uncertainties. It is appropriate to acknowledge that some patients perceive symptomatic benefits from cannabis, particularly for chronic pain and sleep, while also explaining that current evidence does not support reliable opioid-sparing in the acute postoperative setting and that heavy cannabis use may impair bone health and increase the risk of certain complications [18,41,42,53,57-59]. Transparent communication can help align expectations, support adherence to perioperative recommendations, and foster trust in the therapeutic relationship [14,45].

Limitations of the evidence and future directions 

The evidence base regarding cannabis use in orthopaedic surgery is limited by several recurring methodological challenges that warrant careful consideration, and that should inform the design of future studies [4,14,31,45-46,53]. First, most available human data derived from retrospective observational studies often use administrative claims databases or institutional registries. While these sources provide large sample sizes and enable detection of uncommon complications, they also rely on diagnostic codes to identify cannabis exposure and outcomes. Cannabis-related codes frequently capture cannabis use disorder rather than casual use and may underestimate the true prevalence of use [14,31]. Conversely, coding practices may vary by institution and provider, leading to misclassification [14,31]. Outcome definitions, such as infection, nonunion, or thromboembolism, are similarly subject to coding errors and lack standardized clinical validation across studies [14,31].

Second, confounding is pervasive and often incompletely addressed. Cannabis use correlates with younger age, male sex, lower socioeconomic status, comorbid psychiatric conditions, and higher rates of tobacco, alcohol, and other drug use, all of which independently influence orthopaedic outcomes [14,31,45]. Studies that fail to adjust for these factors may overestimate or misattribute risk to cannabis [14,31]. Notably, analyses that distinguish cannabis-only users from those who also smoke tobacco or use multiple substances frequently find that cannabis-only use is not independently associated with higher complication rates, whereas dual use and polysubstance exposure confer the greatest risk [8,17,22-24,48]. However, residual confounding remains unavoidable even in well-designed observational studies [14,31].

Third, exposure characterization is crude in most orthopaedic studies. Details about dose, frequency, duration of use, product composition, and route of administration are rarely available. Without such information, it is impossible to determine dose-response relationships, compare inhaled and oral formulations, or isolate the effects of tetrahydrocannabinol versus cannabidiol-dominant products [4,14,31,53]. Preclinical data suggest that cannabidiol and CB2-selective agonists may promote bone formation and fracture repair, while high-dose tetrahydrocannabinol or cannabis smoke may impair bone or wound healing [33,35,39,40]. Current clinical studies are not equipped to test these hypotheses [4,14,31].

Fourth, bone healing outcomes are incompletely characterized. Few studies incorporate standardized radiographic scoring systems, time-to-union metrics, or bone metabolism biomarkers [4,7,22,53]. Many rely on surrogate markers such as billing codes for nonunion surgery or prolonged immobilization, which may reflect surgeon practice patterns as much as biologic healing [14,31]. Prospective registries with protocolized imaging and clinical assessments would provide more robust data on union, malunion, and functional recovery in cannabis-using patients [4].

Fifth, randomized controlled trials of cannabinoids in orthopaedic surgery are scarce and underpowered. Existing trials often enroll heterogeneous surgical populations, use disparate dosing regimens, and focus on short-term pain endpoints without long-term follow-up [46,50,56-59]. Few trials stratify by baseline cannabis use status or incorporate mechanistic endpoints such as quantitative sensory testing, inflammatory biomarkers, or imaging-based markers of bone remodeling [46,50,56]. High-quality trials tailored to specific orthopaedic procedures, such as long bone fracture fixation, spine fusion, or arthroplasty, are needed to clarify whether any cannabinoid formulation provides clinically meaningful analgesia or influences healing [46,50,56].

Future research should therefore prioritize well-designed prospective cohort studies and randomized or pragmatic trials that address these limitations. Key features should include objective verification of cannabis exposure using biochemical measures where feasible, detailed assessment of dose, route, and product composition, separation of cannabis-only users from those with polysubstance exposure, and systematic capture of both bone healing and patient-centered outcomes [4,14,31,45,46,53]. Studies examining cannabidiol-dominated or CB2-selective interventions as adjuncts to fracture healing are of particular interest, given their promising preclinical data [33,35,38-40,52,60,61]. Finally, health services research should explore how cannabis-related policies and patient preferences influence care pathways, adherence, and long-term functional outcomes in orthopaedic populations [1-3,31,47].

In addition to limitations inherent to the underlying literature, this review has several methodological limitations. As a narrative review, we did not perform a formal systematic review or meta-analysis, and we did not quantitatively pool effect sizes or assess statistical heterogeneity across studies. Our search strategy was intentionally broad to capture the rapidly evolving and heterogeneous body of literature on cannabis use in orthopaedic surgery, which may have resulted in the inclusion of studies with variable methodological quality. Furthermore, many topics addressed in this review are supported by a limited number of observational studies, often with inconsistent exposure definitions and outcome measures, restricting the strength of causal inference. Finally, while we summarize key findings and methodological considerations, we did not independently reanalyze primary datasets, and readers should interpret conclusions in the context of these constraints.

## Conclusions

Cannabis use is common among patients undergoing orthopaedic care and is relevant to bone healing, perioperative analgesia, and postoperative outcomes. Overall, current evidence does not support routine perioperative cannabis use as an opioid-sparing strategy, and heavier or inhaled use may be associated with less favorable healing or complication profiles in some patients. Orthopaedic teams should perform routine preoperative screening, counsel patients on the avoidance of smoked and vaporized products around fracture fixation or arthrodesis, and prioritize validated multimodal analgesic approaches. Prospective research that better characterizes cannabis exposure and orthopaedic-specific outcomes is needed to guide future recommendations. 
